# Improving early audiological intervention via newborn hearing screening in Belgium

**DOI:** 10.1186/s12913-018-2878-3

**Published:** 2018-01-30

**Authors:** Bénédicte Vos, Christelle Senterre, Michel Boutsen, Raphaël Lagasse, Alain Levêque

**Affiliations:** 1 Research Center Epidemiology, Biostatistics and Clinical Research, School of Public Health, Université libre de Bruxelles (ULB), Route de Lennik 808, 1070 Brussels, Belgium; 2 Research Center Health Policy and Systems–International Health, School of Public Health, Université libre de Bruxelles (ULB), Route de Lennik 808, 1070 Brussels, Belgium; 3 Centre d’Epidémiologie Périnatale (CEpiP), Newborn Hearing Screening Program Agency, Route de Lennik 808, 1070 Brussels, Belgium; 4 Agence InterMutualiste (IMA), Avenue de Tervueren, 188/A, 1150 Brussels, Belgium

**Keywords:** Program assessment, Newborn hearing screening, Hearing impairment, Audiological intervention

## Abstract

**Background:**

Newborn hearing screening programs aim to lower the ages at audiological intervention among hearing-impaired children. In Wallonia and Brussels (Belgium), audiological intervention data are not collected in the screening program, and the ages at initiating audiological care have never been assessed. This study aimed to assess the evolution in the ages at initiating audiological intervention in the context of a newborn hearing screening program implementation.

**Methods:**

This population-based descriptive study used data from the Belgian healthcare billing database. The main outcomes were the children’s ages at the initial audiological assessment, hearing-aid fitting, and cochlear implantation. Results were compared to the same outcomes from another Belgian regional program (Flanders) that was implemented one decade earlier. Annual birth cohorts from 2006 to 2011 were included in the study.

**Results:**

In Wallonia-Brussels, the median ages for all outcomes tended to decrease over time but remained higher than in Flanders for each birth cohort. For all outcomes except the hearing-aid fitting, differences in median ages between the two regions became less pronounced during the study period. In 2006, < 23% of the children from Wallonia-Brussels received any audiological care before the age of 12 months and these proportions were approximately 2-fold greater in the subsequent birth cohorts. For all outcomes, early care (< 12 months) was typically delivered less frequently in Wallonia-Brussels, compared to the delivery in Flanders. These region-specific differences exhibited a decreasing trend over time, and statistically significant differences were less common in the later birth cohorts.

**Conclusions:**

We conclude that the hearing screening program in Wallonia and Brussels promoted earlier audiological intervention among hearing-impaired children. However, milestones recommended by experts for an early intervention were not totally encountered. We also recommend collecting audiological intervention data as part of this program, which can facilitate more accurate and regular program evaluation.

## Background

Hearing impairment affects 1–3 children per 1000 newborns in the well-baby nursery population and 2–4% of infants who are admitted to neonatal intensive care units [1]. Compared to other congenital affections, such as phenylketonuria, galactosemia, or congenital hypothyroidism, hearing impairment is one of the most prevalent conditions in newborns [2]. All these affections can be screened at birth and newborn hearing screening programs have been largely implemented across the world [3, 4]. These programs are designed to early identify hearing-impaired children and consequently early enrol them in intervention programs. They have successfully rjeduced the age of hearing impairment identification among children [5–7], which leads to lower ages at enrolment in intervention programs and hearing-aid fitting [8]. In contrast, infants who were not screened were approximately 2 years older at their diagnosis and hearing-aidfitting, compared to infants who underwent screening [5, 6]. Thus, the Joint Committee on Infant Hearing recommends hearing screening before the age of 1 month, in order to identify hearing impairment before the age of 3 months of age and enrol the children in an intervention program as soon as possible (preferably before the age of 6 months) [9]. Most screening programs have targeted these milestones, and have been able to improve early audiological intervention among hearing-impaired children.

Early audiological intervention provides remarkable benefits for hearing-impaired children, as early identified hearing-impaired children show better language outcomes, compared to late-identified children [10–13]. These benefits may include achieving language (receptive and expressive), speech, and vocabulary skills that fall within the normal limits for hearing peers. The children’s social-emotional development was also enhanced in case of early intervention [11]. Although “early” intervention varies from < 3 to < 12 months in the studies, early intervention is associated with better outcomes, regardless of the cut-off age [10, 12, 14–16]. Other factors can also affect the child’s language outcomes: high family involvement, higher mothers’ levels of education and greater maternal self-efficacy are associated significantly with children’s higher language outcome [10, 17, 18].

In Belgium, the French- and Flemish-speaking areas are the political authorities for preventive medicine and they exhibit differences in their implementation of preventive healthcare programs, including newborn hearing screening. In the French-speaking area (the Wallonia-Brussels Federation), the hearing screening program was implemented at the end of 2006, and is only routinely monitored for its screening process. The program does not collect data regarding audiological care outcomes, such as enrolment in intervention services, hearing-aid fitting, or cochlear implantation [19], and conclusions cannot be made regarding the effect of this screening program on the age at initiating audiological intervention. In contrast, the Flemish-speaking area (Flanders) has a hearing screening program that was implemented in 1998, and this program routinely reports data regarding the ages at diagnosis and intervention [20]. The present study aimed to evaluate the change in age at initiating audiological intervention among hearing-impaired children after the implementation of the Wallonia-Brussels screening program. We hypothesised that the implementation of the screening program would lower the ages at initiating audiological intervention, and we compared the Wallonia-Brussels data to the Flanders data, as the Flanders program was implemented almost one decade earlier than the Wallonia-Brussels program, and showed conclusive outcomes.

## Methods

### Study design and database considerations

As the Wallonia-Brussels screening program did not collect data regarding audiological care outcomes, this population-based descriptive study used data from the Belgian healthcare billing database (National Sickness Fund). This database is managed by a public agency (*Intermutualiste Agency*), and includes data regarding billed healthcare and administrative information from all seven public health insurers in Belgium. The healthcare data are identified using Belgian billing codes, and the *Intermutualiste Agency* extracted all relevant data for 2006–2011 in June 2015, which allowed us to analyse annual birth cohorts and ages at initiating audiological intervention.

### Outcomes

The three main study outcomes were the ages at the initial audiological assessment, hearing-aid fitting, and cochlear implantation. In Belgium, the initial audiological assessment is performed at the patient’s enrolment in the intervention centre. All three events have distinct billing codes. For the present study, we also used a fourth outcome that was defined as the first instance of any audiological care (i.e., initial assessment, hearing-aid fitting, or cochlear implantation). The billing database does not include medical diagnosis or hearing screening-related outcomes, which made it impossible to examine outcomes that were directly related to the hearing impairment diagnosis and/or screening outcome.

We selected all relevant billing codes that were in place in 2006 or during the following years. The codes for an initial audiological assessment were widely reported in the database because they may be used for children with other disabilities that are associated with a transiently elevated hearing threshold (e.g., otitis media). Therefore, to only include children with permanent hearing impairment in this outcome, we only considered cases with both an initial assessment code and a code for hearing-aid fitting and/or cochlear implantation. Consequently, children who were not provided with hearing technology were not included in the initial assessment outcome (e.g. some children with unilateral or mild hearing loss).

The children’s age for each outcome was calculated in months (as a truncated value), based on their date of care and date of birth. If several codes were used for a single event in the same child (e.g. two cochlear implants were placed at different times), we only considered the first code. We considered the first event in the ‘any audiological care’ outcome.

### Inclusion criteria

This study evaluated annual birth cohorts from 2006 (the first available year in the billing database) to 2011. Children were included if they underwent audiological care (initial assessment, hearing-aid fitting, or cochlear implantation) at < 3 years of age to ensure a standardised comparison, as the different birth cohorts had unique follow-up periods. This cut-off was selected because it was reported as the age at diagnosis without a hearing screening program [5, 8]. The use of a 3-year period would allow us to capture children with neonatal impairments that were or should have been detected by the screening program and it would limit the inclusion of children with hearing impairments acquired later during childhood. We excluded birth cohorts after 2011 to avoid any potential overestimation of early intervention. Finally, 685 children composed the study population (289 in Wallonia-Brussels and 396 in Flanders).

### Statistical analyses

Children were grouped according to their birth region (Wallonia-Brussels [French-speaking] vs. Flanders [Flemish-speaking]) using the ZIP code for their residence at birth, in order to associate them to the relevant regional screening program. The outcomes according to birth cohort and region were calculated as median ages and percentiles (25th and 75th), and trend lines for median values by region were fitted to these results using Microsoft Excel. Next, we analysed the outcomes according to the proportions of children who received the four types of audiological care (any audiological care, initial assessment, hearing-aid fitting, and cochlear implantation) before the age of 12 months (“early” care) or between the ages of 12 and 36 months (“late” care). The Joint Committee on Infant Hearing recommends initiating intervention before the age of 6 months [9] but very few of the children in our database fulfilled this criterion; thus, we extended the cut-off for “early” care to < 12 months. The outcomes were analysed using the Wilcoxon-Mann-Whitney test to evaluate differences in distributions and the chi-square (or Fischer’s test, where applicable) to evaluate differences in proportions. All data management and statistical analyses were performed using SAS software (version 9.2).

This study was performed based on the legal mandate of the *Agence Intermutualiste* (Program Act 24/12/2002), and received its approval; the agency performed the analyses for each individual’s data and aggregated the data for this study.

## Results

### Median ages at the initiation of audiological intervention

**Fig. 1:**
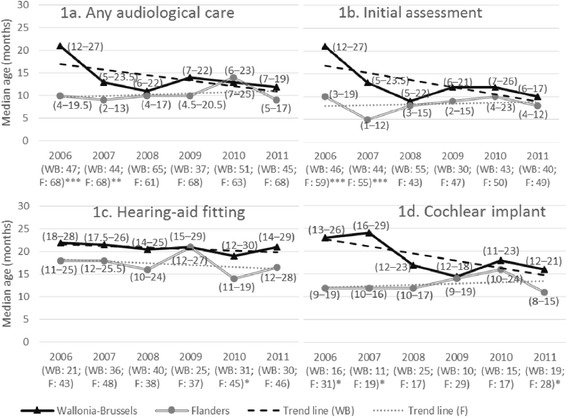
Median age (P25-P75 under brackets) according to birth cohort (2006–2011) and region. X-axis: group size according to region. F: Flanders; WB: Wallonia-Brussels; *P*-values: * < 0.05; ** < 0.01; *** < 0.001

The median ages for any audiological care, initial assessment, and cochlear implantation showed a large initial decrease in Wallonia-Brussels, with some increases in intervention age in the 2009–2010 cohorts, and a resumption of the downward trend in 2011. In particular, the median ages at the initial assessment exhibited a large decrease from 21.0 months (P25–P75: 12.0–27.0 months) in 2006 to 9.0 months (P25–P75: 5.0–22.0 months) in 2008. The any audiological care outcome exhibited a similar trend, and the age at cochlear implantation initiated a large decrease 1 year later, from 24.0 months (P25–P75: 16.0–29.0 months) in 2007 to 14.5 months (P25–P75: 12.0–18.0 months) in 2009. In contrast, outcomes were more constant over time for the longer-standing program in Flanders, but the Flanders cohort also showed some increased ages at intervention in 2009–2010 (Fig. 1).

For each birth cohort and outcome, the median ages were higher in Wallonia-Brussels, compared to the ages in Flanders, with two exceptions (hearing-aid fitting in 2009 and any audiological care in 2010). For example, in Wallonia-Brussels, half of the hearing-impaired children underwent any audiological care before approximately 21 months in 2006, and although this improved to approximately 12 months in the last cohorts, children in Flanders benefitted from an earlier intervention (half of them underwent any audiological care at approximately 10 months) (Fig. 1).

Unlike the decreasing trends that we observed in Wallonia-Brussels, the median ages for the various outcomes remained relatively constant in Flanders. Therefore, the differences in the various outcomes between the two regions were generally greater in the earlier birth cohorts (statistically significant differences), and these differences became less pronounced over time. The exception to this trend was the hearing-aid fitting outcome. For the same outcomes, the distributions (P25–P75) typically overlapped between Brussels-Wallonia and Flanders, with the exception of cochlear implantation in 2007. Results were based on small size groups (Fig. 1).

Considering the median ages, delay between initial assessment and hearing-aid fitting was shorter in Wallonia-Brussels than in Flanders in most birth cohorts, while delay between initial assessment and cochlear implantation was shorter in Flanders (Fig. 1).

### Proportions of audiological care performed before the age of 12 months

**Fig. 2:**
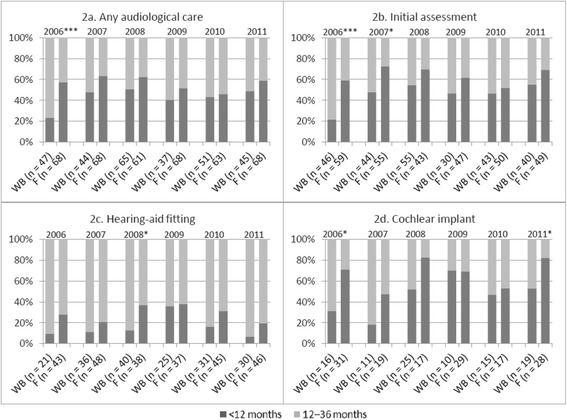
Proportion of children undergoing ‘early’ vs ‘late’ audiological care according to birth cohort and region. F: Flanders; WB: Wallonia-Brussels; *P*-value: * < 0.05; ** < 0.01; *** < 0.001

Less than 23% of the children in the 2006 cohort from Wallonia-Brussels received any audiological care or underwent an initial assessment before the age of 12 months. These proportions evolved greatly in the subsequent birth cohorts (approximately 2-fold greater). A significant increase was also observed after the 2008 cohort in the proportions of children undergoing early (< 12 months) cochlear implantation. In contrast, the proportions of early initial assessment were approximately 50–70% in Flanders, and the proportions of early hearing-aid fitting were approximately 2-fold greater than those in Wallonia-Brussels, with the exception of the 2009 cohort. In Flanders, the proportions of early care did not exhibit any major changes over time, even if the proportions of early cochlear implantation exhibited variability over time (Fig. 2).

Early care for all four outcomes was typically delivered less frequently in Wallonia-Brussels, compared to the delivery in Flanders. One noticeable exception is that the proportions of early hearing-aid fitting and cochlear implantation were quite similar between the two regions in 2009 (approximately 36% for hearing-aid fitting and 70% for cochlear implantation). Nevertheless, these region-specific differences exhibited a decreasing trend over time, and statistically significant differences were less common in the later birth cohorts (Fig. 2).

## Discussion

The present study aimed to assess the change in age at the time of initiating audiological intervention in the context of the newly implemented Wallonia-Brussels hearing screening program, using data from a population-based healthcare billing database. We compared those findings to the data from Flanders, which has an established newborn hearing screening program. For the first time since the screening program implementation, this study assesses the age of children at audiological intervention in Wallonia-Brussels. Outcomes have not been monitored in the program, which is major oversight that precludes any assessment of the program’s effectiveness. The absence of audiological care-related data also raises questions regarding the continuation of care and tracking of infants with positive screening results, which are recommended in public health programs [21, 22].

In Wallonia-Brussels, audiological intervention was initiated at an increasingly earlier age during infancy throughout the various birth cohorts. In contrast, this intervention was typically initiated at an earlier age in Flanders. Our findings exhibited relatively stable ages for the various outcomes in Flanders, which may be related to the relatively established nature of the Flanders program. Consequently, the age gaps between outcomes in Wallonia-Brussels and Flanders tended to decrease over time, especially for the any audiological care, initial assessment and cochlear implantation outcomes, which may be related to the Wallonia-Brussels program “catching up” to the Flanders program. When considered together, our findings suggest that the newborn hearing screening program exerted a positive effect on the age at initiating audiological intervention among hearing-impaired children. These improvements must be monitored to determine whether the Wallonia-Brussels outcomes continue to become closer to the Flanders outcomes and the targets that are recommended by the Joint Committee on Infant Hearing [9]. We cannot definitively conclude that there is a direct causal link between the screening program and outcome improvements, as we cannot discount the possible positive effects of professional training, parental involvement, or care delivery. Nevertheless, it is likely that the early screening was largely responsible for the outcome improvements, based on the importance of screening programs that has been demonstrated in previous studies [6, 7].

A children’s initial assessment could be considered as their effective enrolment in a hearing intervention program, which is an important outcome to monitor in this field. The median ages for this outcome in the present study were above the recommended age of < 6 months [9], with only 25% of the included children in Wallonia-Brussels achieving this milestone. These results are worse than those from screened children in previous studies, but they are also better than those from unscreened children [5, 6], which is to be expected. Thus, to achieve an assessment age of < 6 months in Wallonia-Brussels, we recommend the implementation of a tracking database and a well-organised trajectory of care to facilitate enrolment in the intervention program. Furthermore, cochlear implantation and hearing-aid fitting were performed at a relatively late age for a large subset of children in Wallonia-Brussels, despite the fact that the median ages at cochlear implantation were lower than those in other programs [23, 24]. For example, hearing-aids were fitted at > 20 months in approximately 50% of the children in the present study, which is a long delay from the initial assessment ages. Compared to the other outcomes in Wallonia-Brussels, hearing-aid fitting showed the lowest improvement over time. Progressive or mild hearing loss, medical indications recommending hearing-aid versus cochlear implantation or individual audiological evolution may explain the delay in hearing-aid fitting in some children. Further studies are need to explain this late fitting and absence of real improvement over time, which will likely require more detailed data regarding hearing screening and hearing-impairment outcomes to examine the individual’s trajectories of care and health data of hearing-aid fitted children.

The data showed that the introduction of the newborn hearing screening program in Wallonia-Brussels reduced the age at audiological intervention until a plateau in performance at a less than satisfactory level, and we observed several delays in initiating audiological intervention and few early interventions among hearing-impaired children. We cannot make any definitive conclusions regarding the cause(s) of these delays in Belgium, but previous studies in other contexts have found that delayed interventions may be related to parental or professional factors, administration of healthcare and hearing services, additional disabilities, or financial barriers (e.g., no insurance coverage) [25–28]. Most of these factors may be relevant in Belgium, except financial barriers should not be a contributing factor, as hearing-aids and cochlear implants are completely reimbursed for hearing-impaired children. Other factors such as turnover of professionals and lack in their training, lack of parents’ awareness about normal child’s hearing evolution or about importance to perform the hearing tests, organisation of care and possible delay in medical appointments may be encountered in Belgium and may explain this plateau in our results. Another issue might be failure in the hearing screening program, with newborns either not being screened or not being tracked and followed-up in cases with positive screening results. For example, in 2006, 10.5% of the newborns were not tested and 4.5% of the newborns were not tracked and properly followed-up in the screening program in Wallonia-Brussels and around 6% of the newborns were not tested in the screening program in Flanders in 2004 [19, 20]. Additional studies are needed to precisely identify the factors that affect the early initiation of audiological intervention in Belgium and to adapt consequently the organisation of children’s care.

We encountered methodological limits due to our study design. First, as most studies are designed using the Joint Committee on Infant Hearing’s age cut-off (< 6 months) [9], it is difficult to compare their findings to our findings. The 1-year cut-off in the present study was essential, given that only very low proportions of children in Wallonia-Brussels received audiological care before the age of 6 months. This cut-off did favourably bias our analysis of early intervention in Wallonia-Brussels, as a large proportion of children initiated audiological intervention between the ages of 6–12 months. Second, we used successive annual birth cohorts to examine the effect of the hearing screening program (which was launched in 2007), but some birthing facilities were performing hearing testing before 2007 (for all newborns or for high-risk neonates) and other facilities implemented the screening after 2007. The 2007 cohort should not be considered a clear demarcation point in the screening implementation, but it is important to consider our results in the context of the screening program’s gradual implementation. Third, we allocated children based on the ZIP code of their residence at birth. This approach cannot exclude the fact that children from Flanders were analysed as children from Wallonia-Brussels, and vice versa. For example, all children from Brussels were included in the French-speaking program, although Brussels is a bilingual area and some children should be considered from the Flemish program [29]. Merging the healthcare billing database and the regional newborn hearing screening program databases would have improved the accuracy of our results by providing additional information regarding the administration and results of the hearing screening tests. The anonymised screening data from Wallonia-Brussels made this approach impossible. Fourth, we used a 3-year follow-up period in the present study, which allows the inclusion of cases in which the impairment appeared during early-infancy, because postnatal hearing loss may concern up to 20–5% of the children with permanent hearing impairment [30–32]. So, without access to medical records, we cannot exclude the potential inclusion of hearing loss acquired after the neonatal period that are not targeted by the newborn screening program assessment when interpreting our results. However, the standardised 3-year follow-up period increases the validity of our comparisons across the various birth cohorts, which is a strength of this study.

This study design also included other important strengths. First, our data were population-based and representative of care that was delivered, as Belgium provides universal access to healthcare. Second, our study included hearing-impaired newborns who were missed by the screening program at birth but subsequently identified during the 3-year follow-up period (e.g., false negative results or loss to follow-up). This is not typical of other studies that evaluated hearing screening programs, as they typically evaluated data from the screening program itself, with sometimes insufficient follow-up period. Third, our design used population-based data that can be routinely collected via healthcare billing, which indicates that this approach is a potentially low-cost option for monitoring audiological intervention among hearing-impaired children.

We recommend accurately assessing the long-term success of the screening program. To that end, we recommend enhancing the data collection in the Wallonia-Brussels screening program with information regarding basic outcomes at the enrolment in intervention services, hearing-aid fitting, and cochlear implantation. If enhancement of data collection is not possible due to legal or organizational issues, we recommend using at least named and medical data from the intervention services or other sources, in accordance with professional secrecy or privacy concerns, to monitor the long-term effects of the screening program.

## Conclusions

Early detection and intervention among hearing-impaired children is the main purpose of newborn hearing screening programs. Given the absence of data on audiological intervention in the Wallonia-Brussels screening program in Belgium, we used data from a healthcare billing database to evaluate the success of this program. Our findings indicate that the median ages for initiating audiological intervention, and the proportions of children who received early audiological care, improved after the implementation of the screening program. In Wallonia-Brussels audiological intervention was typically initiated at a later age than in Flanders, where the screening program was implemented a decade earlier. The database that we used was not designed for analysing audiological outcomes among hearing-impaired children, which raises several methodological questions. Therefore, we strongly recommend developing a method or database for collecting accurate data regarding screening and audiological data in hearing-impaired children, as these data are needed to perform accurate and ongoing evaluations of the Wallonia-Brussels hearing screening program.

## References

[1] Erenberg A, Lemons J, Sia C, Trunkel D, Ziring P (1999). Newborn and infant hearing loss: detection and intervention. American Academy of Pediatrics. Task force on newborn and infant hearing, 1998-1999. Pediatrics.

[2] Kaye CI, Accurso F, La Franchi S, Lane PA, Hope N, Sonya P (2006). Newborn screening fact sheets. Pediatrics.

[3] Sloot F, Hoeve HL, de Kroon M, Goedegebure A, Carlton J, Griffiths HJ (2015). Inventory of current EU paediatric vision and hearing screening programmes. J Med Screen.

[4] Williams TR, Alam S, Gaffney M (2015). Progress in identifying infants with hearing loss-United States, 2006-2012. MMWR Morb Mortal Wkly Rep.

[5] Durieux-Smith A, Fitzpatrick E, Whittingham J (2008). Universal newborn hearing screening: a question of evidence. Int J Audiol.

[6] Sininger YS, Martinez A, Eisenberg L, Christensen E, Grimes A, Hu J (2009). Newborn hearing screening speeds diagnosis and access to intervention by 20-25 months. J Am Acad Audiol.

[7] Halpin KS, Smith KY, Widen JE, Chertoff ME (2010). Effects of universal newborn hearing screening on an early intervention program for children with hearing loss, birth to 3 yr of age. J Am Acad Audiol.

[8] Weichbold V, Nekahm-Heis D, Welzl-Mueller K (2006). Ten-year outcome of newborn hearing screening in Austria. Int J Pediatr Otorhinolaryngol.

[9] Joint Committee on Infant Hearing (2007). Year 2007 position statement: principles and guidelines for early hearing detection and intervention programs. Pediatrics.

[10] Moeller MP (2000). Early intervention and language development in children who are deaf and hard of hearing. Pediatrics.

[11] Yoshinaga-Itano C (2004). Levels of evidence: universal newborn hearing screening (UNHS) and early hearing detection and intervention systems (EHDI). J Commun Disord.

[12] Fulcher A, Purcell AA, Baker E, Munro N (2012). Listen up: children with early identified hearing loss achieve age-appropriate speech/language outcomes by 3 years-of-age. Int J Pediatr Otorhinolaryngol.

[13] Boons T, Brokx J, Frijns J, Philips B, Vermeulen A, Wouters J (2013). Newborn hearing screening and cochlear implantation: impact on spoken language development. B-ENT.

[14] Vohr B, Jodoin-Krauzyk J, Tucker R, Johnson MJ, Topol D, Ahlgren M (2008). Early language outcomes of early-identified infants with permanent hearing loss at 12 to 16 months of age. Pediatrics.

[15] Meinzen-Derr J, Wiley S, Choo DI (2011). Impact of early intervention on expressive and receptive language development among young children with permanent hearing loss. Am Ann Deaf.

[16] Vohr B, Jodoin-Krauzyk J, Tucker R, Topol D, Johnson MJ, Ahlgren M (2011). Expressive vocabulary of children with hearing loss in the first 2 years of life: impact of early intervention. J Perinatol.

[17] Stika CJ, Eisenberg LS, Johnson KC, Henning SC, Colson BG, Ganguly DH (2015). Developmental outcomes of early-identified children who are hard of hearing at 12 to 18 months of age. Early Hum Dev.

[18] Yoshinaga-Itano C, Sedey AL, Wiggin M, Chung W (2017). Early hearing detection and vocabulary of children with hearing loss. Pediatrics.

[19] Vos B, Lagasse R, Leveque A. The organisation of universal newborn hearing screening in the Wallonia-Brussels Federation. B-ENT. 2013;(Suppl 21):9–15. 24383218

[20] Van Kerschaver E, Boudewyns AN, Stappaerts L, Wuyts FL, Van de Heyning PH (2007). Organisation of a universal newborn hearing screening programme in Flanders. B-ENT.

[21] Raffle A, Gray M (2007). Screening: evidence and practice.

[22] Muse C, Harrison J, Yoshinaga-Itano C, Grimes A, Brookhouser PE, Epstein S (2013). Supplement to the JCIH 2007 position statement: principles and guidelines for early intervention after confirmation that a child is deaf or hard of hearing. Pediatrics.

[23] Fitzpatrick EM, Johnson E, Durieux-Smith A (2011). Exploring factors that affect the age of cochlear implantation in children. Int J Pediatr Otorhinolaryngol.

[24] le Roux T, de Swanepoel W, Louw A, Vinck B, Tshifularo M (2015). Profound childhood hearing loss in a South Africa cohort: risk profile, diagnosis and age of intervention. Int J Pediatr Otorhinolaryngol.

[25] Holte L, Walker E, Oleson J, Spratford M, Moeller MP, Roush P (2012). Factors influencing follow-up to newborn hearing screening for infants who are hard of hearing. Am J Audiol.

[26] Larsen R, Munoz K, DesGeorges J, Nelson L, Kennedy S (2012). Early hearing detection and intervention: parent experiences with the diagnostic hearing assessment. Am J Audiol.

[27] Armstrong M, Maresh A, Buxton C, Craun P, Wowroski L, Reilly B (2013). Barriers to early pediatric cochlear implantation. Int J Pediatr Otorhinolaryngol.

[28] Serville MN, Demanez L, Demanez JP (2004). Diagnosis of hearing impairment: factors of delay. Acta Otorhinolaryngol Belg.

[29] Vos B, Lagasse R, Levêque A (2014). Main outcomes of a newborn hearing screening program in Belgium over six years. Int J Pediatr Otorhinolaryngol.

[30] Weichbold V, Nekahm-Heis D, Welzl-Mueller K (2006). Universal newborn hearing screening and postnatal hearing loss. Pediatrics.

[31] Watkin PM, Baldwin M (2011). Identifying deafness in early childhood: requirements after the newborn hearing screen. Arch Dis Child.

[32] Young NM, Reilly BK, Burke L (2011). Limitations of universal newborn hearing screening in early identification of pediatric cochlear implant candidates. Arch Otolaryngol Head Neck Surg.

